# Effectiveness of training of spiritual intelligence components on depression, anxiety, and stress of adolescents

**Published:** 2015

**Authors:** M Ebrahimi, Z Jalilabadi, KH Ghareh Chenagh, F Amini, F Arkian

**Affiliations:** * Clinical Psychology, Islamic Azad University, Science and Research Branch, Tehran, Iran; ** Clinical Psychology, Islamic Azad University, Sari Branch, Iran; *** Clinical Psychology, Islamic Azad University, Karaj Branch, Iran; **** Educational Psychology, Islamic Azad University, Central Tehran Branch, Iran; ***** Educational Psychology, Islamic Azad University, Saveh Branch, Iran

**Keywords:** spiritual intelligence, depression, anxiety, stress, adolescents

## Abstract

**Objective:** As the development of adolescence is identified by different types of stress and youths are further exhibited because of bodily, psychic and cultural issues, this research tried to examine the training efficacy of spiritual intelligence parts on depression, tension, and pressure of teenagers.

**Methodology:** The present study was undergone in the initial part of the educational year 2014-2015. Moreover, it was quasi-empirical via post test-pretest, which employed a checking team. Therefore, forty of the large schoolman scholars in Tehran chose to use the utility sampling approach and registered in the test team, overlooking the group randomly (n = 20). Both groups were pretested by using a demographic survey, rate of grief, stress, and anxiety DASS-42. Eventually, the test team rose for 8 gatherings following the practice of spiritual intelligence elements and the checking team obtained no interruption. Next, both teams were post-tested, and the obtained data were analyzed by using presumed and circumstantial analytical approaches conducted through SPSS21.

**Results:** The sequences showed that the exercise of the spiritual intelligence parts clearly decreased grief, stress, and anxiety in youths.

**Conclusion:** The research found that because of the clear stage of the efficacy of spiritual intelligence factors training, its inexpensive and great acceptability by the youths, particularly while working in a team, it had an immense direct effect on the decrease of grief, stress, and anxiety.

## Introduction

Based on the definition given by WHO (2009) [**26**], adolescence encompasses the years between 11 and 19. This age group faces considerable physical, psychological, and social changes in the development from childhood to adulthood. Furthermore, pressure factors in this age period have significant effects on the emergence of psychological disorders [**11**]. Hence paying a particular attention to factors that can have a positive influence on the psychological states, a decrease of psychological distress and improvement of physical and mental health in adolescents is of great importance.

Stress affects personal relationships and brings physical changes and unstable feelings in this age period, which can lead to a variety of diseases in youths. Being blue is one of the most ubiquity and debilitating psychological matters in adolescence. It has such comprehension prevalence among mental diseases that it was named psychological cold [**12**]. Based on the related accompanied researches in Iran, about 20% of the Iranian students deal with depression. For instance, Zargham indicated in a research in 1996, in an age group in the range of 13-17, that 78% of the female and 75% of the male students have mild to critical depression [**11**]. Depressed adolescents are extremely sensitive, and making a difference between depression, mood swings, and signs of other disorders in them is tough. Depressed adolescents sense that they are not useful, attractive, and valuable. Moreover, they feel that they have no future, and they cannot reach their goals [**4**,**5**]. The existence of such psychological moods in adolescence can be deemed as groundwork for defeat and falling behind progress. Hence, paying a particular attention to depression in adolescence is of exceptional value.

Anxiety is another type of general disease in adolescence, its prevalence being reported to be about 10 to 20 percent (Kandal et al., 2010). Anxiety at the beginning of adolescence leads to the appearance of irritation in interpersonal connections and a decrease of adjustment. Therefore, anxious adolescents have more psychological problems and physical disorders compared to their peers [**20**,**21**]. A survey on “Prevalence of Anxiety Disorders” confirmed that 5-10 percent of the adolescents deal with one of the distinctive patterns of anxiety disorders, which misadjusts the normal life and daily function. Also, anxiety is accompanied by problems such as the reduction in self-esteem, sensing of inadequacy, bankruptcy, and poverty [**7**-**9**]. In addition, the use of variables on psychological disorders in adolescents is critical.

Also, stress is another prevalent factor regarding the psychological issues among adolescents. In other words, stress refers to experiencing states that are considered hazardous for the physical or mental welfare of the person. There have been many investigations about the prevalence of stress in adolescents [**2**]. Bahri Yousef (2010) [**27**] showed that prevalence of anxiety among pupils is of about 26.1%, which is a relatively high number. Narimani and Ariapouran (2007) [**22**] explained in their research that familial stress factors, personal stress portions, and scholarly stress factors have the greatest impact on weight in adolescents, sequentially, among the socio-psychological stress portions. Attending studies on stress and its factors among youths is important due to the sensation associated with young people and according to the high level of prevalence of anxiety among adolescents.

Therefore, according to what has been said, paying a special attention to the performance of psychological interventions is important because it can provide the groundwork for the reduction of related disorders among adolescents. Spiritual intelligence is among the most prominent aspects of human existence, which has a tight connection with many aspects of health, growth, and evolution [**13**]. Regarding the definition of human dimensions of existence, WHO [**26**] points to the spiritual dimension in addition to social, psychological, and physical aspects (Moshiri & Vashyst, 2014). Spiritual intelligence relates to human’s capability to feel something between us and the society we live in concurrently and seamlessly [**16**]. Furthermore, it appeals to the chance of acquiring more knowledge and intelligence as well as providing the required knowledge to reach perfection and progress in life [**13**]. Many investigations showed that there was a relation among spiritual intelligence and various features of one’s well-being [**13**,**16**]. Furthermore, there was a vital connection among spiritual intelligence and the psychological reduction issues in adolescents (Saber Amanian, 2014). Therefore, it predicted that training the components of spiritual intelligence has a significant impact on the decline of psychological diseases such as depression, anxiety, and pressure in youths. Hence, the requirement of handling a precise research was explained. On the other hand, based on the conducted studies in Iran, there has been no relevant research on the effectiveness of the components of spiritual intelligence on the decline of psychological diseases such as depression, anxiety, and pressure in youths. Therefore, to address this research gap in Iran and also the development of mental health in youths, such an examination seems to be required. According to the above-mentioned information, the present research investigates the efficacy of the exercise of the spiritual intelligence parts on stress, anxiety, and depression, on adolescents.

## Methodology

The current study is a quasi-test via posttest-pretest that used a checking team. The people involved were all male high school scholars in Tehran in the first half of the scholarly year 2014-2015. Due to the fact that the least sample community in the empirical studies should be of 15 individuals, a 15-individuals sample size was chosen for each of the groups. Afterwards, in order to improve the statistical power and to control the decreased number of members, a 20-individuals sample size (n = 20) was chosen for each of the samples. The sampling was based on convenience sampling approach using all the male high school pupils in Tehran.

The inclusion measures of the existing research were informed satisfaction and willingness to participate in the research, ability to take part in sessions and cooperation in doing assignments, compliance to help in completing instruments, physical and psychological balance, and the age range between 14 and 18. Furthermore, the exclusion criteria for this research included the following items:

- Lack of enthusiasm to take part in the study and the absence at more than three sessions in the preparation method.

- Lack of capacity to take part in the sessions.

- Lack of ability to cooperate in performing jobs and getting any training and psychological way out of the program and schedule set in the analysis.

The instruments used in the present research consisted of a demographic survey, rate of stress, anxiety, and depression DASS-42.

The demographic questionnaire: this questionnaire was formulated to obtain the private data of participants. Characteristics such as age, gender, education, and marital status were asked in the questionnaire.

Depression, and pressure rate (DASS-42): anxiety, depression, and stress rate (DASS-42) were developed in 1995 by Lovibond [**18**,**19**]. This scale consisted of two forms. The short-form consisted of 21 items that evaluated each of the variables of stress, anxiety, and depression according to seven expressions. The way of responding was based on 4-choice questions (0 = never to 3 = so much). The item distribution was the following: 14 questions for depression, 14 questions for anxiety, and 14 questions for stress. The long-form consisted of 42 parts (14 variables for each one). This scale was based on Likert scale containing none, low, medium, and high values. The least score for each subject was zero and the maximum one was three. Ultimately, the sum of the scores of stress, depression, and anxiety were calculated for each case. The reliability of the scale was determined to be 0.91, 0.84, and 0.90 for the variables of stress, anxiety, and depression by sequentially using Cronbach’s alpha method. The short-form of the scale was approved by Sahebi et al. (2005) for the Iranian society and the inherent unity of this scale was 0.77, 0.79 and 0.78 by using Cronbach’s alpha process. Furthermore, Hanifi, Bahraminezhad, Mirza Khalil Abadi, and Taran [**6**] managed a research to determine the safety of the scale of 0.95, 0.89, and 0.99 for the anxiety, stress, and depression, individually. 

The Statistical Package for Social Sciences (SPSS-21) software was utilized to analyze the obtained data. The survey data were examined according to the descriptive statistics, indices of medium standard deviation, rate, and frequency percentage used. Further, the Analysis of Covariance (ANCOVA) was performed based on inferential statistics.

**Table 1:** Cognitive-behavioral group therapy training protocol

Session	Subject
1	Implementation of pre-test, member familiarity with each other, members’ familiarity with the overall structure of the meetings, defining the feature of spiritual intelligence and its components, notification of stress and stress responses, muscle relaxation and slow breathing training.
2	Benefits of spiritual intelligence and its components and the improvement of daily interactions, in training relaxation and slowing breathing of the students.
3	Description of the relationship between thoughts and psychological well-being in training mental imagery exercises to the participants, training the entrance of students in the spiritual states of consciousness, training muscle relaxation and slow breathing.
4	Training effective coping styles, training ability to garnish daily activities with a sense of holiness, practicing relaxation.
5	Training effective coping responses, mental imagery, and induction, training the capability to use intellectual resources to solve problems in everyday life, relaxation, and slow breathing exercises.
6	Training them to manage anger and stress, training the participants regarding the ability to use virtue behaviors (forgiveness, gratitude and abundance), to practice relaxation and slow breathing.
7	Assertiveness training, positive interpersonal relationships, autonomy, learning and practicing the use of virtue behavior in everyday life, relaxation and slow breathing exercises.
8	Training and teaching a personal management plan and environmental mastery to the participants of the program, to sum up the training sessions before the end of the meeting, to take a posttest and to appreciate participants, to take a posttest from participants.

**Research Findings**

The average and standard deviation of the years of participants were 16.48 ± 1.10, and all the participants were males. The descriptive statistics related to the average and nominal deviation of the spiritual intelligence and public health in the two groups according to the pretest and posttest were mentioned in the **Table 2**.

**Table 2:** Descriptive statistics of scores of research variables in the two groups according to the pretest and post test

Component	Index	Experiment		Control	
		Pretest	Posttest	Pretest	Posttest
Depression	Average	15.40	7.30	14.01	13.70
	Standard deviation	2.45	2.08	2.79	2.97
Anxiety	Average	16.35	8.35	14.70	14.65
	Standard deviation	3.61	2.66	1.83	1.95
Stress	Average	17.80	8.85	15.05	15.01
	Standard deviation	2.28	1.75	1.82	1.83

According to **Table 2**, the average scores of the variables of anxiety, stress, and depression decreased in the experimental group compared to the control group.

**Table 3:** Levene test results which investigate the default homogeneity of variances of depression, anxiety and stress in posttest

Variable	Stage	F	Df1	Df2	Sig. level
Depression	Posttest	0.567	1	38	0.454
Anxiety	Posttest	1.324	1	38	0.257
Stress	Posttest	0.084	1	38	0.773

According to **Table 3**, the null hypothesis of equality of variances of the two groups in parameters of anxiety, stress, and depression were approved. In other words, the variances of the two groups were equal to each other for the parameters of anxiety, stress, and depression, and there was no significant difference. Therefore, according to the compliance with the Levine defaults, the results of the hypotheses of the study research were permissible.

**Table 4:** Results of multivariable ANACOVA on the scores of posttest with control of pretest in variables of depression, anxiety, and stress

Test	Value	F	Df	Sig. level	Squared Eta	Power
Pylayy effect	0.848	66.723	3	0.001	0.848	0.95
Wilks Lambda	0.152	66.723	3	0.001	0.848	0.95
Hotelling effect	5.560	66.723	3	0.001	0.848	0.95
Ray's largest root	5.560	66.723	3	0.001	0.848	0.95

As mentioned in **Table 4**, the clear stage of all the tests (P < 0.001) revealed that there were variations between the two groups in at least one of the dependent parameters. According to the squared eta, 0.84 percent of the observed differences among individuals was related to the impact of the independent variable (i.e. intervention method). On the other hand, since the statistical power was equal to 0.95 (greater than 0.80), the sample size was admissible. The results related to the significant difference of each of the dependent variables were mentioned in the following table.

**Table 5:** Results of multivariable ANACOVA regarding the investigation of the training effectiveness of spiritual intelligence parts on anxiety, stress, and depression in posttest

Index	Sum of squares	Df	Mean Square	F	Sig. level	Squared Eta
Depression	408.901	1	408.901	62.160	0.001	0.621
Anxiety	396.910	1	396.910	72.826	0.001	0.657
Stress	378.225	1	378.225	117.279	0.001	0.755

Based on **Table 5**, since p < 0.001, the hypothesis related to the differences between depression, anxiety, and stress between the two groups was approved. Moreover, it expressed a 0.621 percent of change in the score of depression; a 0.657 percent of change in the score of anxiety and a 0.755 percent of change in the score of stress, which were due to the independent variable (training of spiritual intelligence components). Therefore, it expressed that the training of mental intelligence components leads to the reducing of stress, anxiety, and depression in adolescents. 

## Conclusion

According to the present study on the training effectiveness of mental intelligence parts on stress, anxiety, and depression in adolescents, the results indicated that the training of spiritual intelligence components had a significant impact on depression, anxiety, and stress in adolescents. This finding is in line with the studies conducted by Mirzaie Mehr (2014), Boroumandzadeh N and Karimi Sani (2015) [**3**], Ismailie, Ahadi, Delavar and Shafi Abadi (2007) [**1**,**14**] and Shojaee and Soleimani (2015) [**10**].

According to Ribbentrop (cited in Safaie et al., 2010), spirituality has a relationship with health thoroughly. Also, the study of patients with chronic pains and psychological problems indicated that patients who do not trust in God and think that they are alone in such problems have no spiritual experiences and receive no religious support from faith communities. Moreover, they are not considered as a religious-spiritual person and are at a greater risk than other people of losing their health. In fact, it seems that the lack of trust in God and the negative religious coping are in close relationship with the low levels of health and severe experience of pain in these patients. Also, it tried to explain those findings that raised the spiritual intelligence in one’s life, raised more trust in God, helped them perform daily works based on knowledge and awareness, helped them being patient in case of incorrect behavior of others, helped them review the day-to-day operations at the time of rest and also helped them leave bad experiences which resulted in providing infrastructures for psychological dynamism and development. Thereby one could be safe from the risk of multiple disorders. According to Wiglizurth, the initiation of the growth of spiritual intelligence leads to the promotion of a variety of skills such as emotional skills. Therefore, they can make themselves more strengthened [**23**-**25**]. Accordingly, Hajian et al. (2012) [**17**] indicated that spiritual intelligence could be used in the prediction of psychological states so that most of the spiritual intelligence scores were considered as predictors for the emotional and psychological empowerment scopes. It was expressed by explaining the finding that promotion of spiritual intelligence, which is obeying God, in fact, results in the improvement of social behavior, respecting mutual feelings and emotions, respecting the rights of others (i.e. strengthening the psychological states), and reducing the psychological issues and problems. Emmons (2000) [**15**] defined spiritual intelligence as one of the most significant factors of emotional, psychological issues and expressed that there was a close link among the psychological and emotional empowerment and spiritual intelligence and personal capabilities and abilities. In this regard, spiritual knowledge helps with promotion, enrichment, and improvement of psychological and emotional skills.

**Acknowledgement**

At this moment, the authors would like to thank the respected authorities of Education and Training Organization in Tehran Province for their assistance. Moreover, we would also like to thank all the participants for their cooperation in the present research. 
